# Hémorragie cérébro-méningée secondaire à une envenimation par morsure de serpent : à propos de deux cas au Centre Hospitalier Universitaire Sourô Sanou de Bobo-Dioulasso, Burkina Faso

**DOI:** 10.48327/MTSI.2022.131

**Published:** 2022-01-17

**Authors:** Pingdéwendé Victor OUEDRAOGO, Catherine TRAORE, Abdoul Aziz SAVADOGO, Wend Pagnangdé Abraham Hermann BAGBILA, Adama GALBONI, Abaz OUEDRAOGO, Ibrahima Stéphane SERE, Athanase MILLOGO

**Affiliations:** 1 Centre hospitalier universitaire Sourô Sanou, Bobo-Dioulasso, Burkina Faso; 2 Université Nazi Boni, Bobo-Dioulasso, Burkina Faso; 3 Université Joseph Ky Zerbo, Ouagadougou, Bobo-Dioulasso, Burkina Faso

**Keywords:** Hémorragie cérébrale, Envenimation, Vipère, Clinique, Hôpital, Bobo-Dioulasso, Burkina Faso, Afrique sub-saharienne, Hemorrhagic stroke, Envenomation, Viperidae, Clinics, Hospital, Bobo-Dioulasso, Burkina Faso, Sub-Saharan Africa

## Abstract

**Introduction:**

Les envenimations par morsure de serpent constituent un problème de santé publique dans les pays en développement. Les complications neurovasculaires sont rares. Nous rapportons deux cas d’hémorragies cérébrales au Centre hospitalier universitaire Sourô Sanou de Bobo-Dioulasso compliquant une envenimation.

**Description clinique:**

La première patiente était âgée de 60 ans avec une gingivorragie et une altération de la conscience. La seconde, âgée de 50 ans, présentait une hémorragie digestive et une hémiplégie droite. La tomodensitométrie (TDM) cérébrale a trouvé des saignements cérébro-méningés chez nos deux patientes. Elles ont reçu de l’antivenin polyvalent Afrique de Vins Bioproducts Ltd ainsi qu’un traitement symptomatique. L’évolution a été favorable sur le plan vital mais avec des séquelles à type de tétraparésie chez la première patiente et d’hémiparésie chez la seconde.

**Discussion - Conclusion:**

Les hémorragies cérébrales sont des complications rares des envenimations par morsure de serpent. Cependant, elles sont responsables d’une morbi-mortalité élevée dans notre contexte.

## Abstract (en)

**Background:**

Envenomation by snakebite is a public health problem in developing countries. Neurovascular complications are uncommon. We report two cases of hemorrhagic stroke at the Sourô Sanou Teaching Hospital in Bobo-Dioulasso, which complicate an envenomation.

**Clinical description:**

The first patient was a 60-year-old woman with no history, who had been admitted 2 hours after snakebite for gingivorrhagia and consciousness disorders. Clinical examination found coma (GCS 7/15) and tetraparesis. The second patient was a 50-year-old woman with no history, who had been admitted one week after snakebite with digestive bleeding. Clinical examination found right hemiplegia and meningeal syndrome. Both patients presented hemorrhagic stroke on brain CT-scan but hemostasis assessment was normal. They received polyvalent antivenom and symptomatic treatment. The outcome was favorable for survival but with sequelae like tetraparesis for first patient and hemiparesis for second patient.

**Discussion - Conclusion:**

The hemorrhagic strokes are rare complications of snake envenomation. Venomous toxins can directly lead to the rupture of intracranial vessels apart of abnormalities of hemostasis. However, the morbidity and mortality related to envenomation by snake bite remains high in our environment.

## Introduction

Les envenimations par morsure de serpent constituent un problème de santé publique négligé dans de nombreux pays de la région tropicale et subtropicale. Chaque année, il se produit 1,8 à 2,7 millions d’envenimations avec 81 410 à 137 880 décès et environ trois fois plus d’amputations et d’incapacités définitives [20]. Au Burkina Faso, 114 126 cas d’envenimation ont été enregistrés de 2010 à 2014 dont 62 293 hospitalisations et 1 362 décès [10]. Les familles de serpent les plus venimeuses sont les élapidés et les vipéridés [23]. Les complications neurologiques secondaires aux morsures de serpent sont dues à l’effet toxique du venin. Elles dépendent des différentes protéines contenues dans celui-ci. Ainsi, les venins d’élapidés (cobras et mambas) entraînent une action toxique directe sur la transmission de l’influx nerveux au niveau des synapses. Les complications neurologiques des envenimations par vipéridés sont la conséquence d’une prédominance des activités pro-coagulantes ou anticoagulantes à l’origine d’accidents vasculaires cérébraux ischémiques ou, plus souvent, hémorragiques [7]. Ainsi, Aissaoui et al [1] ont rapporté une association de lésions cérébrales thrombotiques et hémorragiques lors d’une envenimation grave par la vipère à cornes du Sahara. Cependant, le diagnostic, la surveillance des saignements intracrâniens ainsi que la prise en charge sont difficiles dans notre contexte où les ressources sont limitées.

Nous rapportons deux observations cliniques d’hémorragie cérébrale en rapport avec une envenimation par morsure de vipère au Centre hospitalier universitaire Sourô Sanou.

## Observation 1

**Figure 1:**
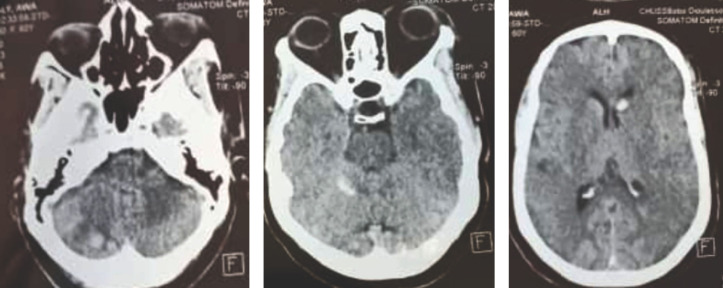
Tomodensitométrie cérébrale montrant de multiples saignements intracrâniens au niveau occipital, cérébelleux et ventriculaire Brain computed tomography showing multiple bleeds in occipital, cerebellar and ventricular regions

Il s’agit d’une patiente de 60 ans, résidant en zone rurale, sans antécédents pathologiques particuliers qui a été victime le 28/11/2020 aux environs de 21 heures d’une morsure de serpent au niveau du gros orteil droit alors qu’elle se rendait aux toilettes. Trente minutes après, elle a présenté des gingivorragies et une altération de la conscience. Dans la formation sanitaire périphérique, une bi-antibiothérapie faite de ceftriaxone 2 g et de métronidazole 500 mg lui a été administrée. Elle a été admise à l’hôpital environ 2 heures après. L’examen clinique a noté une tension artérielle à 110/80 mmHg, un pouls à 109 pulsations/minute, une température à 38,9 °C, une saturation pulsée en oxygène à 89 %, une diurèse à 1 000 ml/24 heures et un œdème du membre inférieur droit remontant jusqu’à la racine. Concernant l’état neurologique, la conscience était altérée avec un score de Glasgow à 7/15 associé à une tétraparésie cotée à 2/5 aux 4 membres. La numération formule sanguine montrait une anémie à 9,1 g/dl et une numération des plaquettes normale à 339 000/mm^3^. La protéine C réactive était à 26 mg/l et la glycémie à 5,8 mmol/l. Le taux de prothrombine était normal à 81 % avec un temps de Quick à 13,8 secondes (temps témoin à 12,5 secondes). Le temps de céphaline activée était normal à 27,9 secondes (temps témoin à 25,4 secondes). L’ionogramme sanguin, la créatine kinase (MB), l’urémie, la créatininémie et les transaminases étaient normaux. Cependant, le taux de fibrinogène, les produits de dégradation de la fibrine et les D-dimères n’ont pu être dosés. La tomodensitométrie (TDM) cérébrale (Fig. 1) a montré de multiples saignements intracrâniens au niveau occipital, cérébelleux et ventriculaire.

La patiente a séjourné un mois dans le service de réanimation avant d’être transférée dans le service de neurologie. La patiente avait reçu l’antivenin polyvalent Afrique de Vins Bioproducts Ltd 3 heures après la morsure de serpent à la dose de 2 ampoules par voie intraveineuse et renouvelée 2 heures après avec arrêt des saignements extériorisés au bout de 4 heures. Aucun effet indésirable n’avait été rapporté. Elle avait bénéficié de mesures générales de réanimation avec oxygénothérapie, sérum salé isotonique, vaccin et sérum antitétanique, paracétamol 1 g/8 heures, néfopam 20 mg/8 heures et des soins infirmiers. Une antibiothérapie à base d’amoxicilline et d’acide clavulanique 1,2 g/8 heures, de métronidazole 500 mg/8 heures avait été administrée pendant 3 semaines et de l’acide tranexamique 1 g en bolus puis 1 g en perfusion de 8 heures. L’évolution a été marquée par une récupération de la conscience et de l’autonomie respiratoire. La patiente a été libérée le 06/02/2021 avec une kinésithérapie. Elle gardait des séquelles à type de tétraparésie après 6 mois de suivi.

## Observation 2

**Figure 2:**
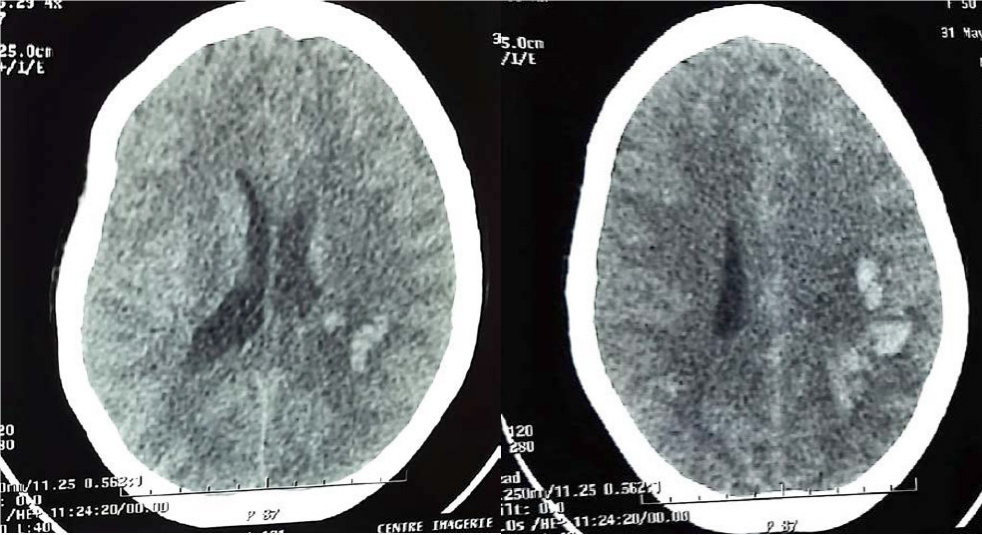
Tomodensitométrie cérébrale montrant une plage hémorragique en fronto-pariétal gauche associée à une hémorragie méningée Brain computed tomography showing bleeds in left fronto-parietal region and subarachnoid hemorrhage

Il s’agit d’une patiente de 50 ans, résidant en zone rurale, sans antécédents pathologiques particuliers, admise dans notre structure le 31/05/2021. Elle avait été victime le 24/05/2021 aux environs de 8 heures d’une morsure de serpent au niveau de la face dorsale du pied gauche, alors qu’elle cherchait du bois en brousse. Elle avait reçu une ampoule d’antivenin polyvalent Afrique de Vins Bioproducts Ltd, 1 g de ceftriaxone et 500 mg de métronidazole dans une structure sanitaire périphérique environ 3 heures après la morsure. L’évolution a été marquée 4 jours après par la survenue de céphalées intenses en casque, associées à des vomissements en jet avec des épisodes d’hématémèse et de méléna. L’examen clinique notait une pression artérielle à 130/70 mmHg, un pouls à 79 pulsations/minute, une température à 38,2 °C, une diurèse à 1 500 ml/24 heures, une plaie punctiforme à la face externe du pied gauche et un œdème du pied et de la jambe gauche. Sur le plan neurologique, la conscience était obnubilée avec un score de Glasgow à 11/15 associé à une raideur méningée et une hémiplégie droite. La numération formule sanguine montrait une anémie à 9,4 g/dl, une hyperleucocytose à 18 820/mm^3^, une numération des plaquettes normale à 322 000/mm^3^ et une glycémie à 6,05 mmol/l. La protéine C réactive était positive à 21 mg/l. Le taux de prothrombine était normal à 81 % avec un temps de Quick à 14,4 secondes (temps témoin à 12,5 secondes). Le temps de céphaline activée était normal à 23,4 secondes (temps témoin à 25,1 secondes). L’ionogramme sanguin, la créatine kinase (MB), l’urémie, la créatininémie et les transaminases étaient normaux. Cependant, le taux de fibrinogène, les produits de dégradation de la fibrine et les D-dimères n’ont pu être dosés. La TDM cérébrale a montré une plage hémorragique fronto-pariétale gauche associée à une hémorragie méningée (Fig. 2). Devant les saignements cérébro-méningés et digestifs, la patiente a reçu une semaine après la morsure, une nouvelle dose d’antivenin polyvalent Afrique de Vins Bioproducts Ltd à la posologie de 2 ampoules par voie intraveineuse, avec arrêt des épisodes d’hématémèse et de méléna au bout d’une heure. Aucun effet indésirable n’a été rapporté. Elle a aussi reçu du sérum salé isotonique, un vaccin et du sérum antitétanique, du paracétamol 1 g/8 heures, du néfopam 20 mg/8 heures, du ceftriaxone 2 g/jour et du métronidazole 500 mg/8 heures pendant 15 jours et des soins infirmiers. L’évolution a été marquée par une amélioration clinique et la patiente a été libérée le 16/06/2021 avec une kinésithérapie. Elle gardait des séquelles à type d’hémiparésie droite après 3 mois de suivi.

## Discussion

Au cours de 3 ans d’expérience dans notre service, nous rapportons deux cas d’hémorragies cérébrales post envenimation par morsure de serpent chez deux patientes sans antécédents pathologiques particuliers, âgées de 50 et 60 ans. Bien que l’espèce du serpent n’ait pas été identifiée par un spécialiste, la description faite par les patientes et/ou leur entourage, la réaction inflammatoire loco-régionale et le syndrome hémorragique sont évocateurs d’une morsure de vipère. Dans une étude réalisée à Ouagadougou, les vipères étaient incriminées dans la plupart des morsures de serpent [8]. En Afrique soudano-sahélienne, cinq espèces sont hautement dangereuses *(Bitis arietans, Echis ocellatus, Echis leucogaster, Cerastes cerastes* et *Cerastes vipera).* L’espèce *Echis ocellatus* est la plus répandue au Burkina Faso [26]. Au Togo, le genre *Echis* était le plus souvent identifié [3]. Les complications neurologiques ne sont généralement rapportées dans la littérature que sous forme de cas cliniques. Dabilgou et al [6] ont rapporté 3 cas d’hémorragies cérébrales post envenimation à Ouagadougou, et Yalcouyé et al [27] ont décrit un cas d’hémorragie cérébrale avec cécité irréversible à Bamako. Les études sur de grandes populations sont rares en Afrique et ne sont pas précises quant aux complications neurologiques. Dans la série de Ouermi et al [21], sur 74 enfants hospitalisés pour morsure de serpent, le diagnostic d’accident vasculaire cérébral n’avait été posé chez aucun patient. Dans l’étude de Touré et al [25], les complications neurologiques (hématome sous-dural et accident vasculaire cérébral) représentaient 2,6 % des patients. Cependant, cette rareté est relative car la plupart des envenimations concernent les populations rurales qui ont le plus souvent recours à la médecine traditionnelle et qui ne disposent pas d’imagerie cérébrale pour diagnostiquer un accident vasculaire cérébral. Dans une revue de la littérature allant de janvier 1995 à octobre 2018, 83 cas d’accidents vasculaires cérébraux sur envenimation ont été publiés avec 20,5 % d’hémorragies cérébrales [2]. En effet, les accidents vasculaires cérébraux hémorragiques et ischémiques sont vus spécialement dans les morsures de vipère [22]. Cependant, l’estimation des effets directs de la neurotoxicité centrale des envenimations peut être difficile. Ainsi, sur les deux patients décrits au Bénin dans un hôpital rural, le diagnostic d’hémorragie cérébrale avait été posé sur la base des signes cliniques et du liquide cérébrospinal [4]. Le transfert des patients dans des hôpitaux de référence et la réalisation de l’imagerie cérébrale devant les troubles de la conscience et les signes de localisation neurologique devraient permettre d’améliorer le diagnostic et la prise en charge des hémorragies cérébrales par envenimation.

Le saignement est dû à une lésion vasculaire induite par le venin et est entretenu par d’autres mécanismes en lien avec le venin tels que la thrombopénie, la coagulopathie de consommation, la défibrination et l’hyperfibrinolyse [15]. Cette coagulopathie a été rapportée par Dabilgou et al [6] avec une élévation des D-dimères à 7 500 mg/l chez un patient, une thrombopénie et une diminution du taux de prothrombine chez un autre. C’était également le cas de Yalcouyé et al [27] dont le patient avait une thrombopénie et un sang complètement incoagulable au test de coagulation sur tube sec réalisé à l’admission. De même, les métalloprotéases zinc-dépendantes, appelées hémorragines, détruisent la membrane basale située sous l’endothélium capillaire à l’origine d’hémorragies locales ou systémiques [18, 24]. Cela pourrait expliquer le saignement chez nos patientes dont le bilan d’hémostase était normal. Il en était de même pour Kumako et al [13] dont les deux patients observés ne présentaient pas de trouble d’hémostase. Cependant, notre plateau technique ne nous a pas permis de doser le taux de fibrinogène et les produits de dégradation de la fibrine. En effet, les toxines des venins de serpent n’activent pas toujours la totalité des facteurs de coagulation mais aboutissent à des concentrations plasmatiques de fibrinogène basses ou indétectables avec des taux normaux de certains facteurs de coagulation [17]. Un seuil critique de 1 g/l de fibrinogène était utilisé pour prédire les saignements importants [16]. L’absence de troubles de l’hémostase est ici atypique au vu des complications hémorragiques rapportées. Pour la première patiente, le premier bilan biologique était normal probablement du fait de sa précocité après la morsure, et nous pouvons supposer que si un second bilan avait été réalisé plus tard, il aurait mis en évidence des anomalies de l’hémostase en lien avec la consommation des plaquettes et des facteurs de coagulation observés habituellement lors d’envenimations compliquées d’hémorragies systémiques. De même, Mion et al [19] ont montré qu’en l’absence de traitement avec de l’antivenin, le délai nécessaire pour obtenir un taux de prothrombine supérieur à 50 % était de 5,8 jours en moyenne et que le taux de céphaline activée pouvait rester au-delà de 1,5 fois la norme durant 4,7 jours en moyenne. Cela pourrait expliquer le bilan d’hémostase normal, réalisé une semaine après l’envenimation chez notre seconde patiente, où les collections hémorragiques étaient encore détectables (en dehors de tout drainage) alors qu’il pourrait ne plus y avoir de saignement actif.

La prise en charge des envenimations dépend de la gravité du tableau clinique. Ainsi, l’envenimation est classée en 5 grades de gravité selon Chippaux [5]. Nos deux patientes ont été classées grade 4 du fait du saignement cérébro-méningé. L’hospitalisation en réanimation est recommandée dans ce cas. Une de nos patientes qui était dans le coma y a été admise. L’immunothérapie antivenimeuse, qui agit globalement sur les différents constituants du venin, demeure l’unique thérapeutique spécifique [14]. Elle doit être mise en œuvre le plus rapidement possible afin d’éviter le décès, mais également de prévenir ou de limiter les séquelles loco-régionales, neurologiques et rénales. Cependant, au Burkina Faso, le coût supporté par le patient reste le principal frein à l’accessibilité de ce traitement et ce, malgré la subvention apportée par l’État dans certaines zones. La posologie initiale préconisée est de 2 ampoules administrées en 20 à 30 minutes dans une perfusion de soluté isotonique à renouveler si les signes d’une envenimation grave persistent [5]. Nos patientes ont reçu de l’antivenin, avec arrêt des saignements extériorisés au bout de quatre heures et une heure respectivement. En cas d’aggravation du tableau neurologique, la surveillance des saignements intracrâniens pourrait se faire par l’imagerie cérébrale. Le retard ou l’absence de ce traitement met en jeu le pronostic vital ou entraîne de lourdes séquelles. Une de nos patientes a reçu de l’acide tranexamique. En effet, celui-ci a démontré une efficacité et une sécurité dans la réduction du risque de décès lié au saignement, quelle que soit l’étiologie [9].

Les complications cérébrovasculaires sont à l’origine d’un tableau clinique sévère et d’une mortalité élevée. Ghezala et al [11] ont rapporté un cas d’accident vasculaire cérébral hémorragique mortel suite à une envenimation par une vipère à cornes en Tunisie. Dans une étude rétrospective portant sur la prise en charge des envenimations au Nigéria, l’atteinte du système nerveux central et le retard d’hospitalisation étaient les facteurs associés à la mortalité. Ainsi, 49 % des patients ayant une atteinte du système nerveux central sont décédés [12]. Nos deux patientes ont survécu au prix de lourdes séquelles chez la première. Cela s’explique par le tableau clinique sévère (coma) et des saignements intracérébraux multifocaux.

## Conclusion

Les complications neurovasculaires des envenimations sont rares mais le plus souvent dramatiques. Les patientes que nous avons décrites ont présenté des saignements diffus intra-parenchymateux et méningés. Cependant, le taux de prothrombine et le temps de céphaline activée étaient normaux. Ce fait inhabituel traduit la particularité de nos deux observations. La prise en charge efficace et rapide de ces envenimations par l’amélioration de l’accessibilité à l’antivenin permettra de réduire cette morbi-mortalité. Il s’avère donc nécessaire de définir et de mettre à disposition un protocole de prise en charge de ces envenimations sévères.

## Liens d’intérêts

Les auteurs ne déclarent aucun lien d’intérêt.

## Contribution des auteurs

Conception de l’étude, recueil des données, rédaction et correction du manuscrit: OUEDRAOGO Pingdéwendé Victor.

Recueil des données, suivi clinique, surveillance thérapeutique: SAVADOGO Abdoul Aziz, GALBONI Adama, OUEDRAOGO Abaz, SERE Ibrahima Stéphane.

Relecture, correction et validation du manuscrit: TRAORE Catherine, BAGBILA Wend Pagnangdé Abraham Hermann, MILLOGO Athanase
